# A Novel Framework for Remote Sensing Image Synthesis with Optimal Transport

**DOI:** 10.3390/s25061792

**Published:** 2025-03-13

**Authors:** Jinlong He, Xia Yuan, Yong Kou, Yanci Zhang

**Affiliations:** 1 School of Computer Science, Sichuan University, Chengdu 610065, China; jinlonghe_dorcy@foxmail.com (J.H.); kouyong@stu.scu.edu.cn (Y.K.); yczhang@scu.edu.cn (Y.Z.); 2 School of Computer Science, Chengdu University of Information Technology, Chengdu 610225, China

**Keywords:** generative adversarial networks, remote sensing images, optimal transport

## Abstract

We propose a Generative Adversarial Network (GAN)-based method for image synthesis from remote sensing data. Remote sensing images (RSIs) are characterized by large intraclass variance and small interclass variance, which pose significant challenges for image synthesis. To address these issues, we design and incorporate two distinct attention modules into our GAN framework. The first attention module is designed to enhance similarity measurements within label groups, effectively handling the large intraclass variance by reinforcing consistency within the same class. The second module addresses the small interclass variance by promoting diversity between adjacent label groups, ensuring that different classes are distinguishable in the generated images. These attention mechanisms play a critical role in generating more realistic and visually coherent images. Our GAN-based framework consists of an advanced image encoder and a generator, which are both enhanced by these attention modules. Furthermore, we integrate optimal transport (OT) to approximate human perceptual loss, further improving the visual quality of the synthesized images. Experimental results demonstrate the effectiveness of our approach, highlighting its advantages in the remote sensing field by significantly enhancing the quality of generated RSIs.

## 1. Introduction

Image synthesis of remote sensing images (RSIs) aims to generate realistic RSIs from input semantic labels. This task falls within the realm of conditional image synthesis, which not only contributes to geographic deepfake detection [1] but also has numerous valuable applications in fields such as natural disaster prediction [2], ground classification [3], and land-cover development [4]. These applications can significantly assist or even replace human labor in performing repetitive and tedious tasks, greatly improving efficiency. For example, NVIDIA’s AI image generation tool, AI Playground [5], can automatically generate images from semantic labels, showcasing the potential of AI-assisted geographic data generation.

Recent advancements in Generative Adversarial Networks (GANs) [6] have led to fierce competition in the pursuit of visual fidelity. In GAN-based image synthesis, some approaches have achieved remarkable results for high-resolution image synthesis, generating clearer textures and richer semantic features in spatial and spectral dimensions.

However, enhancing visual fidelity in remote sensing image synthesis presents unique challenges. Despite the progress made, most image synthesis studies [7,8,9] primarily target natural scenes, overlooking the distinct characteristics and challenges posed by RSIs. This imbalance limits the application and reliability of AI techniques in geographic information systems.

RSIs, typically captured by satellites or unmanned aerial vehicles (UAVs), differ significantly from natural images taken from ground-level perspectives. On the one hand, RSIs contain a wide variety of objects, each with distinct spectral and spatial characteristics, resulting in more diverse and complex feature information compared to natural images. On the other hand, RSIs often exhibit large intraclass variance—where objects within the same class (e.g., farmland) appear very different due to seasonal changes, sensor variations, or environmental factors—and small interclass variance, where objects from different classes (e.g., farmland and grassland) exhibit highly similar visual characteristics. These attributes make it difficult to accurately distinguish between different land-cover types, adding to the challenges of realistic image synthesis.

Given these challenges, conventional GAN-based image synthesis methods [6], which have shown promising performance in natural image synthesis, struggle to achieve effective RSI synthesis. The inherent differences in semantic label distributions and spectral characteristics between RSIs and natural images further exacerbate this performance gap. Therefore, developing a tailored synthesis framework that accounts for the unique attributes of RSIs is essential to improving both the visual fidelity and semantic consistency of generated remote sensing images. Recently, methods such as DiffusionSat [10], CRSdiff [11], and GEOSynth [12] have been developed to generate remote sensing images using diffusion models. These methods introduce complex control mechanisms, transforming the image-to-image generation process into a more sophisticated prompt-based process. While these approaches represent promising directions for advancing remote sensing image synthesis, they also present certain challenges in terms of lightweight deployment and practical application. Compared to these methods, our research focuses on developing a practical and relatively lightweight solution for RSI generation, aiming to balance generation quality with deployment efficiency.

In this paper, we propose a GAN-based method designed specifically for conditional image generation in the context of RSIs. Our method incorporates two novel attention modules within the image encoder and generator. These two modules adapt to data variations, enabling the generation of a diverse set of features for RSI synthesis. Most methods for loss optimization in generative models, such as pixel-wise loss or adversarial loss, often fail to capture the nuances of human perception. These methods primarily focus on pixel-level accuracy or statistical distribution matching, which may result in artifacts, unrealistic textures, or an overemphasis on fine details that are not aligned with human visual preferences. As analyzed in [13], pixel-wise losses, such as L1 or L2 distance, typically fail to account for perceptual similarities between images, often leading to overly smooth or blurry outputs. Additionally, we reformulate the loss function using optimal transport (OT), aligning the generated data distribution with that of RSIs. This improvement results in a more accurate loss function that corresponds with human perception.

The contributions of this article are as follows:

1. We introduce two attention modules within the image encoder and generator, significantly enhancing the diversity of feature categories for RSI synthesis.
2. By utilizing OT, we reformulate the loss function, ensuring that the generated images align more closely with the ground truth in terms of human perception.
3. The experimental results demonstrate that our algorithm outperforms SOTA algorithms in generating RSIs.

## 2. Related Works

GANs have gained significant attention in recent years due to their flexibility and have been used to address various problems, including image synthesis [14,15,16], style transfer [17], super-resolution [18,19], image completion [20], classification [21], and segmentation [22]. GANs can even handle object reconstruction tasks, although scene complexity poses challenges for computer vision solutions.

### 2.1. GANs in Image Generation

The authors of [23] introduced novel ideas for the generator, transforming noise that follows a given distribution into realistic images. The discriminator is trained to estimate the distance between the generated distribution and the noise distribution. If the generated images successfully deceive the discriminator, they become the final output. DCGAN [24], based on a deep convolutional neural network, improves image generation quality. However, GANs still face issues such as training failures and mode collapse [23]. The paper by [25] shed light on problems with the original GAN and introduced the Wasserstein GAN (WGAN), which addressed convergence issues. To meet the Lipschitz continuity required by WGAN, various methods have been proposed, including weight clipping [6], gradient penalty [26], and spectral normalization [27]. Later, Wu et al. [28] adopted the Wasserstein divergence objective, bypassing the Lipschitz approximation problem and achieving better results. Liu et al. [29] proposed WGAN-QC by using the L2 cost to construct the loss function instead of the L1 cost. Despite the ability of various GAN-based methods to generate realistic images as expected, mode collapse or mode mixture problems can still occur [30].

### 2.2. Diffusion Models in Image Generation

Diffusion models have achieved state-of-the-art results across various modalities, including image generation. DiffusionSat [10] is a generative model trained on large, high-resolution remote sensing datasets, using metadata like geolocation for conditioning instead of sparse text captions. This model excels in generating realistic satellite images and performing tasks such as temporal generation, super-resolution, and inpainting, outperforming prior methods. CRS-Diff [11] incorporates a multi-condition controllable framework for RSI generation, including text, metadata, and image-based conditions. Its advanced control mechanisms enhance stability and precision in image generation, making it a powerful tool for downstream tasks like road extraction. GeoSynth [12] synthesizes satellite images by controlling global style through text prompts or geographic locations. Trained on paired satellite imagery and OpenStreetMap data, GeoSynth demonstrates high-quality, diverse image generation with excellent zero-shot generalization.

### 2.3. Labels to Images

Conditional GANs are a type of hybrid model that can also be applied to image-to-image transformation. They often use an encoder–decoder architecture to establish a connection between paired images and differentiate the distribution of decoded images from that of real ones using a discriminator. For example, the method in [31] transforms images between different styles, while SRGAN [18] uses a similar architecture to generate super-resolution images from their low-resolution counterparts. It combines content loss with adversarial loss for optimization. Spatially adaptive normalization (SPADE) [17] uses a generalized approach to incorporate class information into the generation process, preserving spatial information from input semantic maps. The parameters used to shift and scale feature maps are tensors obtained through convolutions. SPADE [17] prevents map information from fading away during the generation process.

### 2.4. Remote Sensing Images (RSIs) and Semantic Segmentation

Attention mechanisms have achieved significant success in semantic segmentation and can generally be divided into two categories. The first category focuses on channel attention, which selects the most informative features along the channel dimension. For example, PAN [32] employs a global attention mechanism to serve as a context prior for channel-wise feature selection, while Attention U-net [33] uses channel attention to regulate the fusion of high-level and low-level features. However, these approaches typically do not enhance feature representations along the spatial dimension for remote sensing images (RSIs). The second category involves self-attention mechanisms, which compute the representation at each spatial position as a weighted sum of features from all other positions [34,35]. This method effectively captures long-range dependencies critical for semantic segmentation. For instance, ref. [34] implemented two self-attention modules to model contextual information along both the channel and spatial dimensions. Despite their advantages, these methods often lead to increased model complexity and computational inefficiency, posing challenges when processing large volumes of RSIs. In contrast, ref. [36] introduced two lightweight attention modules—one for spatial attention and one for channel attention—specifically designed for high-resolution remote sensing image segmentation. The spatial attention module determines where to enhance features, while the channel attention module decides which features to emphasize. The integration of these two modules has been shown to significantly improve segmentation accuracy. Inspired by these advancements, we design similar attention mechanisms for label-to-image generation, aiming to leverage their benefits while addressing the unique challenges presented by RSIs.

## 3. Methods

### 3.1. Algorithm Overview

**Figure 1:**
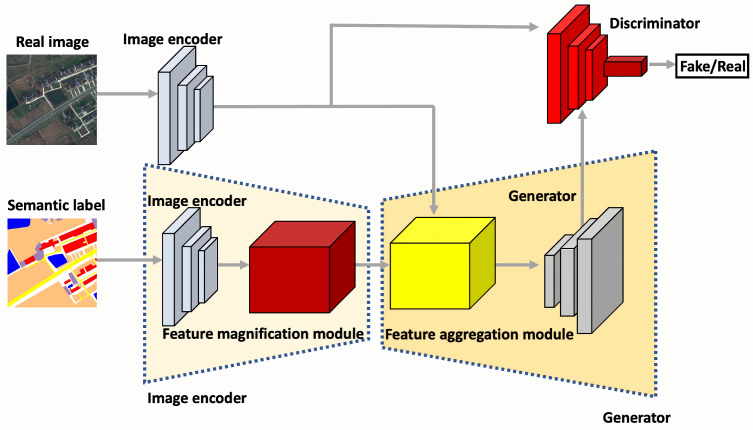
Overall architecture of our proposed GAN-based framework. It consists of an image encoder, a generator, and a discriminator. Two attention modules are added to the image encoder and generator to ensure that the output data are synthesized to more closely resemble RSIs.

As shown in Figure 1, the entire framework follows the GAN architecture, with the encoders responsible for the initial image processing, the generator handling the label-to-image generation, and the discriminator performing adversarial training. Unlike the decoding of RSIs, the processing of labels involves the integration of one attention module into the traditional image decoder, thereby creating an enhanced image decoder, represented by the red module in Figure 1. In the generation phase, to handle diverse feature data from the image encoder, another attention module, represented by the yellow module in Figure 1, contributes to constructing a more robust generator. Finally, the generated distribution and real RSIs undergo adversarial training in the discriminator.

Overall, the proposed method, built upon the standard structure of GANs, has three key optimizations, which are located in the encoder and generator. The first is the feature magnification module, which is concatenated after the image encoder, and the second is the feature aggregation module, which is concatenated before the generator. They work together in the feature space to address the following characteristics of RSIs, which pose unique challenges in RSI synthesis:

- **Small interclass variance:** RSIs are often captured through aerial photography and exhibit minimal variation between different classes. Due to distant viewpoints, pixels from distinct objects tend to appear blurred and similar. This makes it challenging to differentiate them in the feature layer when aligning them with the label data. Image attributes from different categories have similar distributions, such as forest and agriculture in the LoveDA [37] dataset.
- **Large intraclass variance:** RSIs exhibit significant intraclass variance. This means that even within the same label group, objects can appear differently due to varying scenarios and different collection devices. Furthermore, the same class in RSIs often contains uneven elements in addition to distinguishable objects. Thus, a single category can have different distributions in images and feature spaces depending on label position and label structure.

Therefore, the two attention modules serve as data processing blocks in the encoder and the generator. They magnify and aggregate features to represent more distinguishable image features that align with realistic RSIs.

The third optimization is driven by the fact that feature representation is enhanced after processing RSIs. In addition to the conditional adversarial loss, we incorporate the OT metric into the generator to compute perceptual loss, favoring it over feature matching or the VGG network [38].

**Figure 2:**
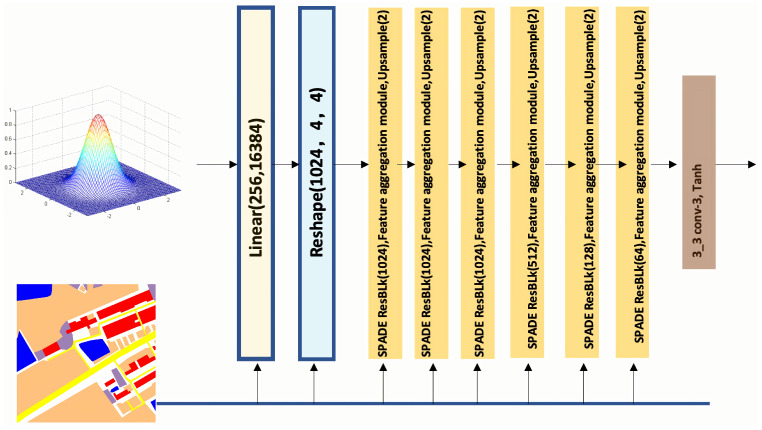
The proposed generator combines SPADE ResBlk [17] with our feature aggregation module to learn rich features.

Our generator is similar to the SPADE generator [17], but the crucial difference is that we integrate a feature aggregation module into every layer of upsampling. This modification enhances our ability to synthesize RSIs. As depicted in Figure 2, our generator includes several ResNet blocks within the upsampling layers.

### 3.2. Feature Magnification Module

**Figure 3:**
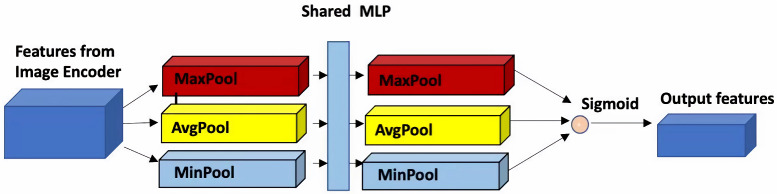
Feature magnification module that transfers image data to a feature space closer to RSIs.

**Figure 4:**
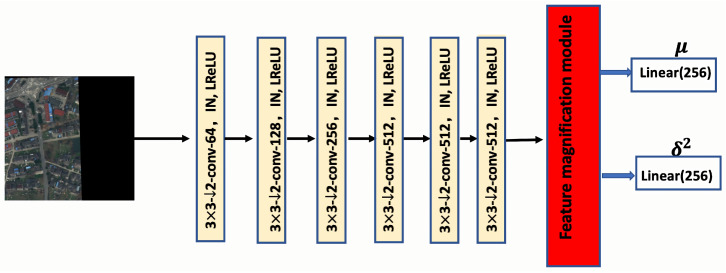
The image encoder of the proposed method. It is constructed using a series of convolutional layers and concludes with the feature magnification module, which outputs linear parameters.

The first attention module, known as the feature magnification module, is integrated into the image encoder for input labels. As shown in Figure 3, this module aggregates multiple sources of information through various channel operations, including global max pooling, average pooling, and min pooling. The outcome is a set of feature descriptors that effectively represent the attributes of RSIs. These feature descriptors then undergo processing via a shared multi-layer perceptron, resulting in richer feature vectors. Finally, these feature vectors are summed element-wise and passed through a sigmoid function. The output of the magnification module is an attention map, which is added to the last convolutional layer of the image encoder, as illustrated in Figure 4.

As mentioned previously, RSIs exhibit large intraclass variance, making it difficult to measure the similarity between the same labels. Feature vectors with similar labels can be unified and mapped across a wide range of feature spaces through the first attention module. As a result, RSI generation maintains uniformity when the input has similar labels.

### 3.3. Feature Aggregation Module

However, feature vectors still face the problem that small interclass variance contributes to a lack of diversity in RSI generation. It is necessary to adapt feature vectors for diversity measurement.

The second attention module is called the feature aggregation module. It resides within the generator and synthesizes more realistic features. Similar to the image encoder, the generator must preserve the attributes of RSIs, where intraclass pixels exhibit higher variance, while inter-class pixels exhibit lower variance. Additionally, the output images should exhibit differences between distinct input labels while maintaining a consistent style within similar or identical label groups.

In this stage, the feature aggregation module focuses on spatial distribution. After pixelation, different objects exhibit unique characteristics. This module takes the feature vectors from the image encoder and aggregates spatial information to learn a weight map. Subsequently, it multiplies this map with the corresponding spatial positions to obtain more representative feature vectors.

**Figure 5:**
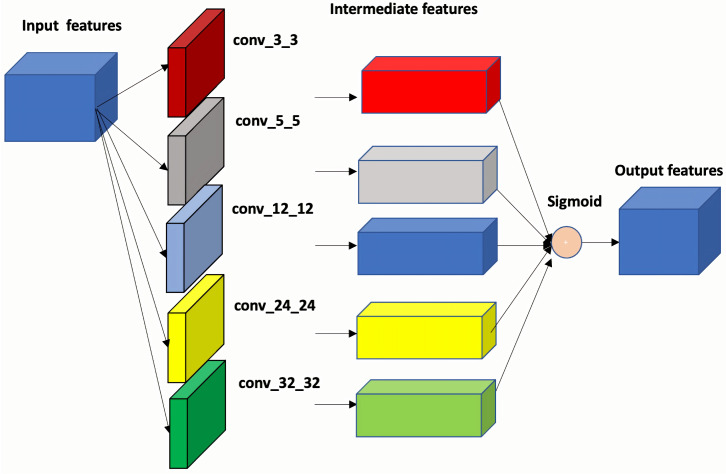
Feature aggregation module that we propose to enrich features.

As shown in Figure 5, the output features from the image encoder are initially processed through several convolution operations with different kernels. These operations generate corresponding feature descriptors, which are then concatenated to create a spatial attention map after using a sigmoid function.

### 3.4. OT Loss

Based on our previous analysis of RSI data, we observed that input labels with less feature expression tend to behave like a uniform distribution or a Gaussian distribution. In our approach, we transform label images from a uniform or Gaussian distribution into a more complicated distribution with multiple feature layers for generated RSIs, just as RSIs are transformed into a discrete set in the feature space. Inspired by Gu’s research [39], we propose a semi-discrete optimal transport method to construct the loss function of the generation process.

**Figure 6:**
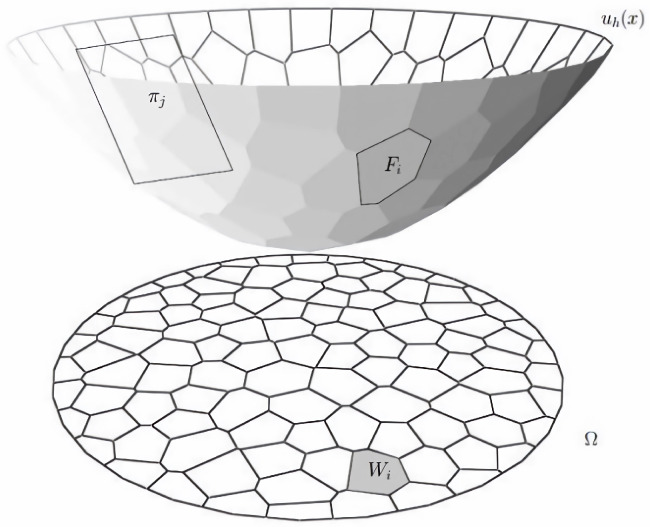
The discrete Brenier potential and optimal transport.

Brenier [40] found the connection between convex geometry and the optimal transport map, which converts the original optimization problem into a gradient map. Considering a semi-discrete transport map T:Ω∼Y, where Ω is a convex domain including the source measure and *Y* is the target domain where the ground truth lies, cell decomposition is carried out in $\Omega \cup_{i=1}^{k}W_{i}$. Each cell $W_{i}$ maps to a target point $\vec{p_{i}}$ via $T:W_{i}∼\vec{p_{i}}$. As shown in Figure 6, each cell $W_{i}$ maps to the slope $\vec{p_{i}}$ of plane $\pi_{i}$. In the context of optimal transport theory, the transport plan $\pi_{i}$ defines the strategy for mapping source features to target features. Brenier’s theorem [40] shows that the piecewise (PL) convex function (Brenier potential), $\{u_{h}\left(x\right):=\max_{i=1}^{n}\left\{\pi_{h,i}\left(x\right)\right\}$, can be applied to find the gradient map, where $\pi_{h,i}\left(x\right)=<x,y>+h_{i}$ is the hyperplane corresponding to $\vec{p_{i}}$, *x* represents the input features, and *y* represents the mapped output features. Thus, the height vector $\vec{h}$ is the only optimizer by minimizing the cost function:

(1) $$E\left(h\right)=\frac{1}{2}\sum_{i=1}^{k}\int_{W_{i}}{\left|\vec{p}-\vec{p_{i}}\right|}^{2}dx$$

Therefore, a better generation strategy should focus more on the philosophy of the global optimum rather than on local mapping between labels and generated RSIs. In our network, let *n* be the number of layers of the generator, let $\vec{F_{i}}$ be the *i*th feature vector of optimal mapping, and let *m* be the number of elements of the feature vector $\vec{F_{i}}$. The OT loss function ${\mathrm{Loss}}_{\mathrm{OT}}$ is defined as

(2) $${\mathrm{Loss}}_{\mathrm{OT}}=\frac{1}{2}\sum_{i=1}^{n}\frac{1}{m}{\left|\vec{F}-\vec{F_{i}}\right|}^{2}$$

On the other hand, WGAN [25] uses weight clipping as the Lipschitz constraint, which has proven to be an effective method for approximating the optimal transport between distributions and improving misconvergence. We propose an alternative weight clipping enforcement as the Lipschitz constraint on the discriminator. Additionally, we take the attention modules into account by imposing another two penalties on the gradient norm for the generated data and real data. The objective is formulated as

(3) $$\begin{array}{c} {\mathrm{Loss}}_{\mathrm{Penalty}}=-\lambda_{1}E_{\vec{F}∼P_{g}}[(∥\nabla_{\vec{F}}D\left(\vec{F}\right){∥}_{2}-1{)}^{2}]-\\ \lambda_{2}E_{\vec{F}∼P_{r}}[(∥\nabla_{\vec{F}}D\left(\vec{F}\right){∥}_{2}-1{)}^{2}] \end{array}$$

where $P_{g}$ and $P_{r}$ are the model distribution and data distribution. The first and second items are the penalties we propose to enforce the Lipschitz constraint. We set $\lambda_{1}=5$ and $\lambda_{2}=5$ as the optimal values by performing a grid search combined with cross-validation, evaluating their joint impact on the validation set. Here, D(∗) is the discriminator, and E is the objective function.

In the end, the generator fine-tunes its model parameters during training by optimizing three key components: conditional GAN loss, OT loss, and discriminator penalty loss. The overall loss function combines the following four components:

(4) $$\min_{G}\left(\max_{D}\left({\mathrm{Loss}}_{D}\right)+{\mathrm{Loss}}_{G}+{\mathrm{Loss}}_{\mathrm{Penalty}}+\lambda_{\mathrm{OT}}{\mathrm{Loss}}_{\mathrm{OT}}\right)$$

where we set $\lambda_{\mathrm{OT}}=3$ as a result of joint optimization with $\lambda_{1}$ and $\lambda_{2}$, using grid search and cross-validation to determine the best combination. *G* is the generator.

## 4. Experiments and Analysis

### 4.1. Experimental Setup

The proposed method was tested on a single GeForce RTX 3090 GPU with the following two datasets:

1. LoveDA [37]: A remote sensing image dataset that focuses on different geographical environments, including urban and rural areas. We train the models with seven semantic classes, including background, building, road, water, barren, forest, and agriculture.
2. Gaofen-2 (GID-15) [41]: A satellite image dataset that consists of 15 semantic classes with low resolution, non-uniform hue, and unstable picture quality.

To evaluate the generative quality, we utilized several metrics to measure the similarity between the generated images and the ground truth. These metrics included the Inception Score (ISC) [21], Kernel Inception Distance (KID) [42], and Frechet Inception Distance (FID) [43]. Additionally, we utilized DeepLabv3+ [44] for semantic image segmentation, evaluating the mean intersection over union (mIoU) between the ground-truth labels and generated semantic labels to assess the quality of the generated images.

### 4.2. Results and Comparison

We compared our method with several state-of-the-art (SOTA) semantic image synthesis methods, including Pix2PixHD [7], SPADE [17], SMIS [45], and INADE [46]. All models were retrained for RSIs. The quantitative scores of the ISC, KID, and FID are presented in Table 1 and Table 2, where lower scores indicate better image synthesis quality.

**Figure 7:**
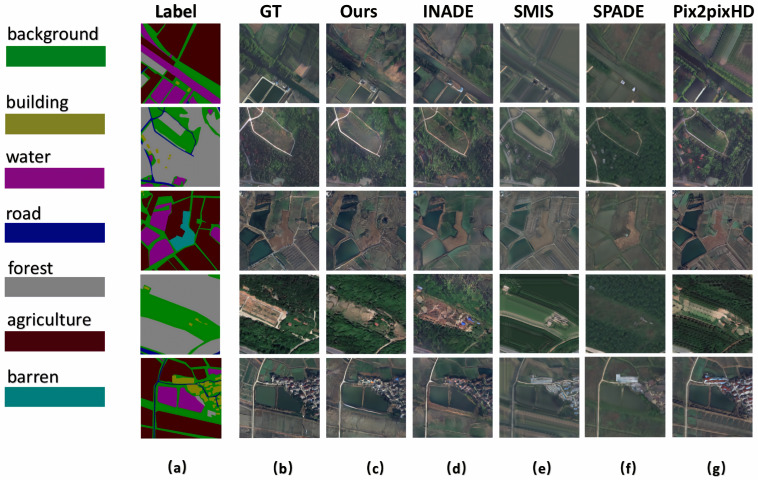
Qualitative comparison with SOTA semantic image synthesis methods on rural images from the LoveDA dataset. The column (**a**) shows the semantic labels, and the column (**b**) represents the ground truth. The column (**c**–**g**) are generated results of our method, INADE, SMIS, SPADE and Pix2PixHD, respectively.

For the LoveDA [37] dataset from Wuhan University, Table 1 shows that the different methods achieved nearly identical ISC scores, making it difficult to distinguish between them. The FID and KID scores reveal that our method excelled in RSI synthesis. Additionally, higher mIoU scores indicate better segmentation results, as shown in Table 3, which demonstrates the superior generative quality of our method. Figure 7 shows various synthesized rural areas, where column (a) shows the input labels, column (b) shows the ground truth RSIs, and columns (c) to (g) present the results of our method, INADE, SMIS, SPADE, and Pix2PixHD, respectively. It can be seen that the results from our method closely resemble the ground truth, while SMIS and Pix2PixHD struggled to synthesize details, resulting in jagged pixels, particularly noticeable in the fourth row of Figure 7.

**Figure 8:**
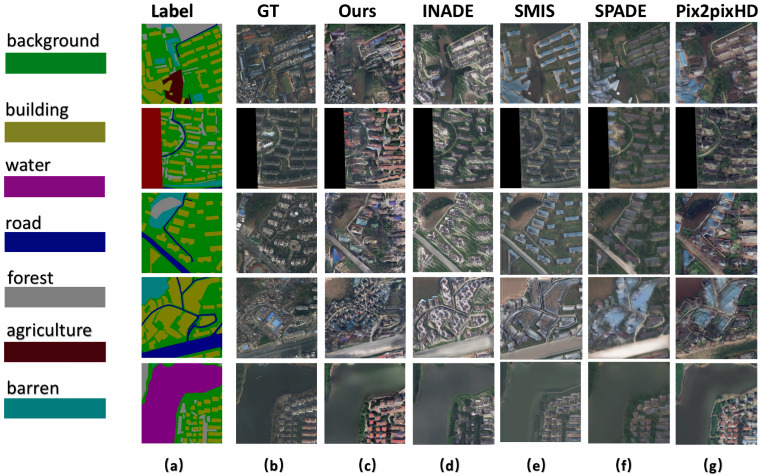
Qualitative comparison of state-of-the-art semantic image synthesis methods on the LoveDA dataset. The column (**a**) shows the semantic label maps, and the column (**b**) displays the corresponding ground-truth images. The column (**c**–**g**) are generated results of our method, INADE, SMIS, SPADE and Pix2PixHD, respectively.

Figure 8 shows the results of RSI generation in urban areas. Our method accurately converted every label category into the corresponding image pixel group, faithfully representing semantic information such as roads, farmland, water, and barren land. Even in urban areas with complex pixel compositions, our method generated RSIs that closely matched the label images and resembled the ground truth in terms of details. For instance, in the fourth row of Figure 8, it can be seen that the SMIS, SPADE, and Pix2PixHD methods failed to generate the building group. The ISC, KID, and FID scores in Table 1 further validate our method’s superior performance for urban images from the LoveDA dataset [37].

When generating large areas with a single label, most SOTA methods failed to accomplish this task, producing inaccurate results. This issue is one of the main problems addressed by our method.

**Figure 9:**
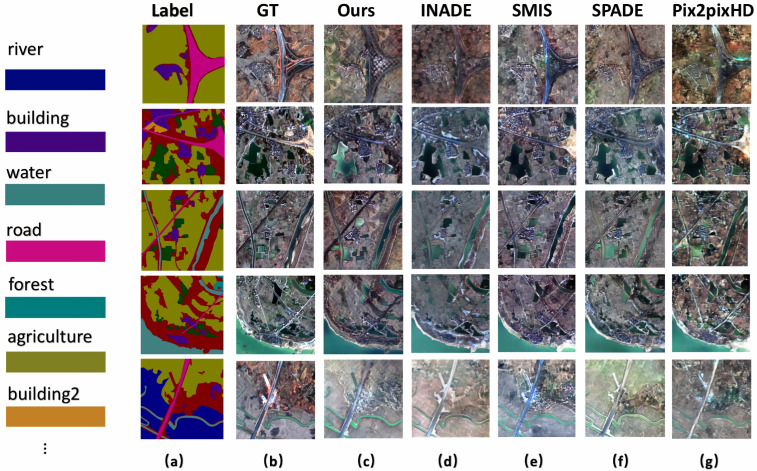
Qualitative comparison of state-of-the-art semantic image synthesis methods on the GID-15 dataset. The column (**a**) shows the semantic label maps, and the column (**b**) displays the corresponding ground-truth images. The column (**c**–**g**) are generated results of our method, INADE, SMIS, SPADE and Pix2PixHD, respectively.

The GID-15 dataset, which includes 15 categories and various hue styles, poses a greater challenge in RSI synthesis. Our method outperformed others in terms of KID scores and performed comparably to INADE, as shown in Table 2. As shown in Figure 9, our method performed comparably to INADE from a visual perspective. However, considering that GID-15 consists of low-resolution RSIs with various hue styles, generating images without incorporating style transfer, as done by INADE, is challenging. Nevertheless, our method demonstrated robustness and effectiveness for RSIs due to the incorporation of attention modules and the OT loss function.

**Figure 10:**
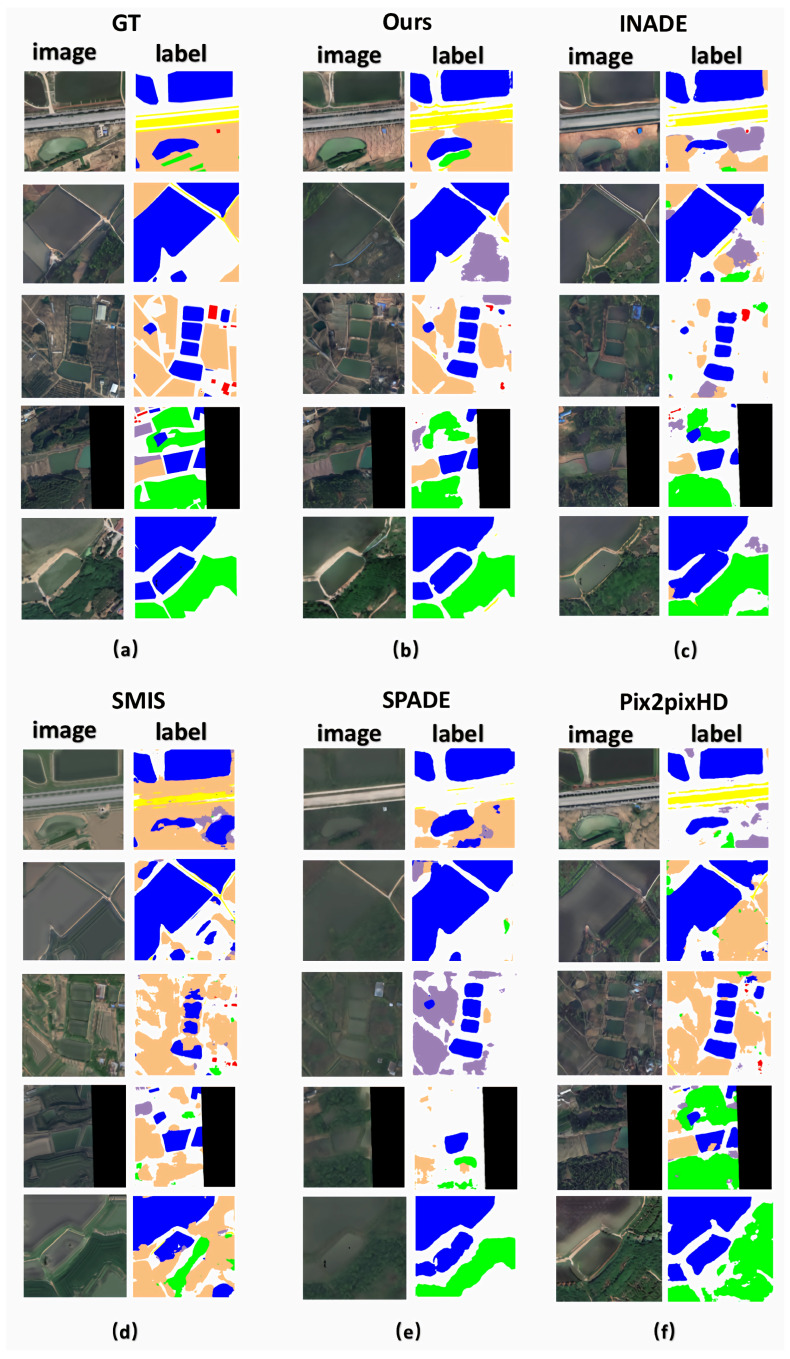
Qualitative comparison with SOTA semantic image synthesis methods and segmentation results on the LoveDA dataset. (**a**) Ground-truth images and labels. (**b**–**f**) Synthesized images and segmentation results from our method, followed by those from INADE, SMIS, SPADE, and Pix2PixHD, respectively.

In this experiment, we employed the classical segmentation network [44] to segment the RSIs generated by various methods, including ours. Based on the fact that high-quality RSI generations should produce close segmentation results to ground truth, we compared these segmentation results to the ground-truth labels. Figure 10 demonstrates that the segmentation results from our method were the closest to the ground truth. Moreover, Table 3 indicates that our method achieved a higher mIoU than the SOTA methods. These findings affirm the superior generative quality of the RSIs produced by our method.

### 4.3. Ablation Study

To verify the effectiveness of our innovations, we conducted ablation experiments on both the attention modules and the OT loss function.

**Figure 11:**
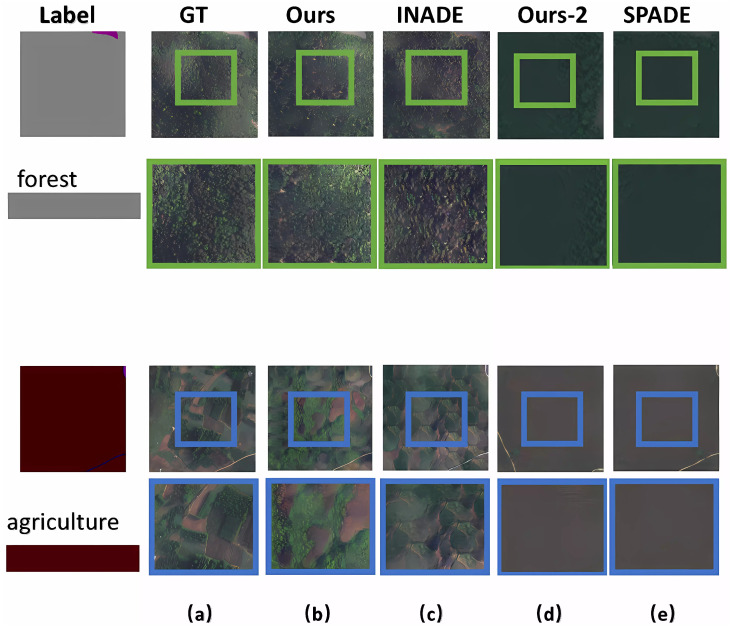
Ablation experiments on attention modules for images with a single label. Column (**a**) shows the ground-truth images. Column (**b**) shows the results from the proposed method. Column (**d**) shows the results from the proposed method without attention modules. Column (**c**) and column (**e**) show the results from INADE and SPADE, respectively.

For the attention modules added to the network, we compared the results from the methods, as shown in Figure 11. While SPADE [17] converted a single category label into a realistic image using spatially adaptive normalization, it struggled to generate target pixels accurately, resulting in jagged edges in large areas with a single label input. However, as shown in Figure 11, the experiments without attention modules exhibited similar limitations to SPADE. Column (d) represents the results of the network without attention modules. Our method with attention modules generated realistic RSIs, meeting the requirement of large intraclass variance. The FID scores in Table 4 also confirm the feasibility and effectiveness of the proposed method.

**Figure 12:**
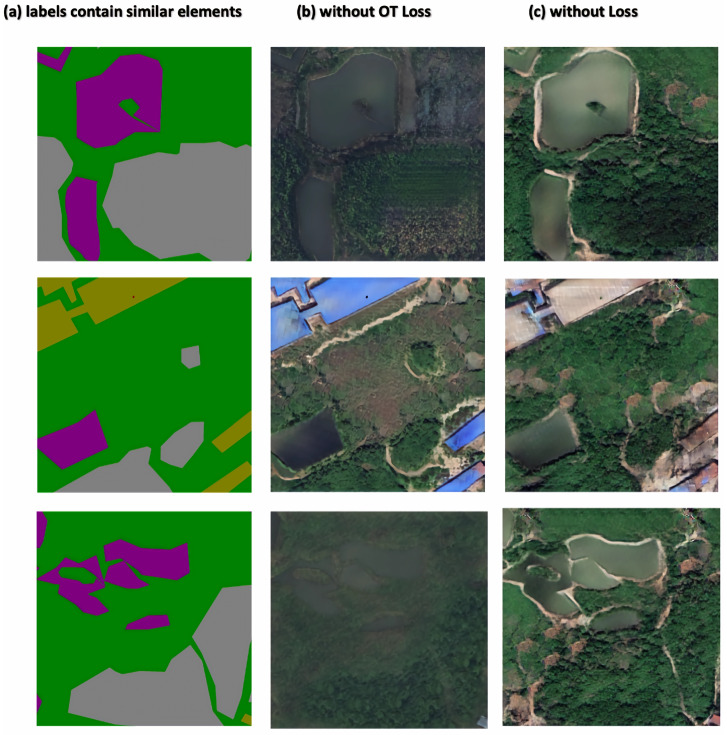
Ablation experiments on the OT loss function. Column (**b**) and column (**c**) show the results from the proposed method with and without the OT loss function, respectively.

Regarding the OT loss function, we demonstrated its necessity with a series of labeled images with similar compositions. Figure 12 illustrates labels with similar elements, which should ideally generate images in the same style, as shown in column (c). However, due to minor imperfections in the training data, they often resulted in images with different colors, textures, and brightness, as seen in column (b). To achieve uniform generative results, the OT loss function constrains data distributions to a stable range at the feature layer. Once all the features are decoded into RSIs, they meet the requirement of small interclass variance. The experiments above illustrate the feasibility and effectiveness of the proposed OT loss function.

As mentioned in Section 3, grid search and cross-validation were used to assess the contribution and balance of the proposed modules within the GAN and to determine their optimal weight parameters. In the original GAN, both the generator and discriminator are assigned an equal weight of 1 when constructing the system. Since our proposed method introduces $\lambda_{1}$, $\lambda_{2}$, and $\lambda_{OT}$, we maintain the same default weight for the generator and discriminator to avoid complicating the optimization problem. We evaluated the impact of different hyperparameter combinations on the accuracy of RSI generation across the training, validation, and testing sets.

**Figure 13:**
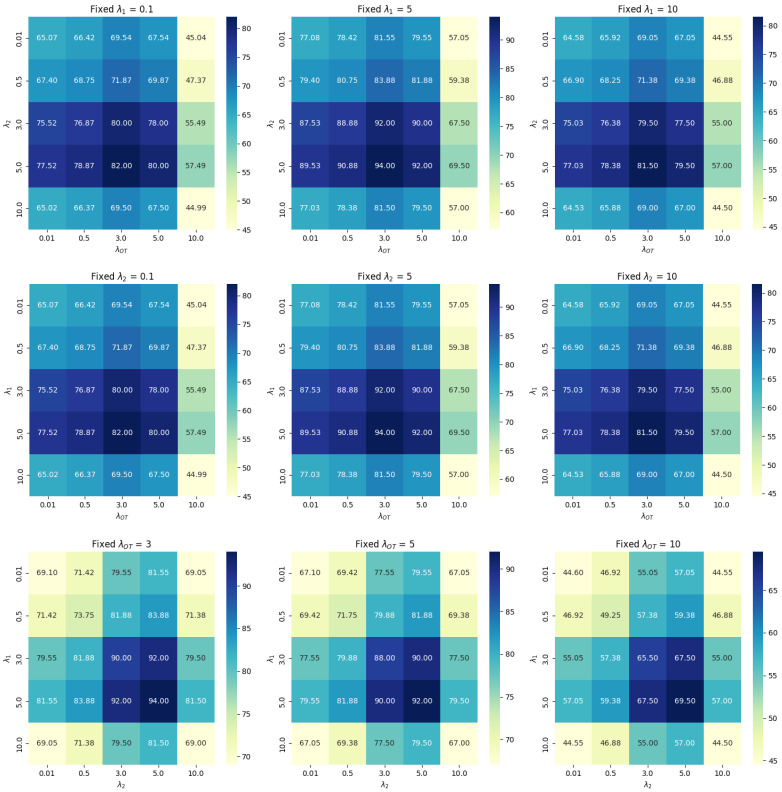
Ablation study on parameter settings: The first row presents the optimal combination of $\lambda_{2}$ and $\lambda_{\mathrm{OT}}$ for a fixed $\lambda_{1}$. The second row presents the optimal combination for a fixed $\lambda_{2}$. The third row presents the optimal combination for a fixed $\lambda_{\mathrm{OT}}$.

Table 5 presents the results of extensive experiments aimed at determining the optimal values for the parameters $\lambda_{1}$, $\lambda_{2}$, and $\lambda_{\mathrm{OT}}$. The findings indicate that the optimal values were $\lambda_{1}=5$, $\lambda_{2}=5$, and $\lambda_{\mathrm{OT}}=3$. To further illustrate the process of selecting these weight parameters, Figure 13 presents heat maps in which one variable is fixed while the optimal values of the other two are determined. This visualization offers an intuitive understanding of how different parameter combinations affect model performance, thereby indirectly validating the effectiveness of our optimal parameter selection process. For instance, when $\lambda_{1}$ is fixed, the first row shows the accuracy for different values of $\lambda_{2}$ and $\lambda_{\mathrm{OT}}$. Similar heat maps, in which either $\lambda_{2}$ or $\lambda_{\mathrm{OT}}$ is fixed, reveal how these parameters interact. Overall, these visualizations help us pinpoint the optimal parameter regions that lead to the best performance, validating our parameter selection process.

## 5. Conclusions

In this paper, we present a novel GAN-based method for remote sensing image synthesis. We introduce two attention modules in the image encoder and the generator, which allow our network to adapt to the unique attributes of RSIs. Additionally, we formulate a loss function based on optimal transport theory to better align our generated images with human perception. Experimental results on various datasets demonstrate the superiority of our method in terms of visual fidelity. Our approach has the potential to advance the field of remote sensing image synthesis and facilitate applications in geographic information systems.

## Figures and Tables

**Table 1:** Comparison of quantitative scores with SOTA methods on the LoveDA dataset.

Dataset	Ours	INADE	SMIS	SPADE	PixPixHD
ISC	4.7683	4.76	4.768	4.7683	4.768
KID	0.0316	0.04983	0.0436	0.05867	0.03796
FID	73.53	95.46	82.10	107.338	87.27

**Table 2:** Comparison of quantitative scores with SOTA methods on the GID-15 dataset.

Dataset	Ours	INADE	SMIS	SPADE	PixPixHD
ISC	6.993	6.993	6.993	6.997	7.093
KID	0.04678	0.05098	0.0530	0.05201	0.04980
FID	170.79	169.43	261.83	210.42	199.86

**Table 3:** mIoU comparison with SOTA methods.

Dataset	Ours	INADE	SMIS	SPADE	PixPixHD
LoveDA	73.53	63.08	45.9	69.98	47.0
GID-15	80.0	75.5	53.76	67.07	39.86

**Table 4:** FID scores from the ablation study on the attention modules.

Dataset	Ours	INADE	SPADE	Ours-2
LoveDA	159.33	167.05	191.27	198.24
GID-15	170.79	169.43	210.42	225.67

**Table 5:** Ablation study on parameter settings for $\lambda_{1}$, $\lambda_{2}$, and $\lambda_{\mathrm{OT}}$.

$\lambda_{1}$	$\lambda_{2}$	$\lambda_{\mathrm{OT}}$	Training Accuracy (%)	Validation Accuracy (%)	Testing Accuracy (%)
0.01	0.01	0.01	85.0	83.0	82.5
0.01	0.01	1	86.5	84.5	84.0
0.01	0.01	5	87.0	85.0	84.5
0.5	0.5	0.5	87.5	85.5	85.0
0.5	0.5	3	88.5	86.5	86.0
1	1	1	89.0	87.0	86.5
1	1	5	90.0	88.0	87.5
3	3	3	92.5	90.0	89.5
5	5	3	94.0	91.5	91.0
3	3	10	93.0	90.5	90.0
10	10	10	88.0	86.0	85.5

## Data Availability

Data are contained within the article.
